# Practice‐Level Variation in the Provision of Subsidised Dental Services to Adult Danes in 2019: A Register‐Based Study

**DOI:** 10.1111/cdoe.13048

**Published:** 2025-06-03

**Authors:** Eero Raittio, Vibeke Baelum

**Affiliations:** ^1^ Department of Dentistry and Oral Health Aarhus University Aarhus Denmark; ^2^ Institute of Dentistry, University of Eastern Finland Kuopio Finland

**Keywords:** clinical decision‐making, dental care, dental practice patterns, health services research, patient safety, quality of health care

## Abstract

**Objectives:**

The aim was to investigate practice‐level variation in common dental diagnostic, preventive, and care services provided for Danish adults who underwent a dental examination.

**Methods:**

This was a nationwide register‐based study. Subsidised dental services delivered during the 13‐week period subsequent to the provision of one of three eligible dental examinations (extended, basic, or recall examination) during the first 9 months of 2019 were investigated. Bayesian multilevel regression models were used to estimate the practice‐level average predicted probability of supragingival care, subgingival care, individual prevention, bitewing radiographs, and endodontic treatment, and the average predicted count of extractions and direct restorations while adjusting for individual sociodemographics and dental treatments received during the previous 10 years.

**Results:**

The final sample included 445 516 examinations conducted in 1593 dental practices. Supragingival care after basic or recall examinations showed the lowest practice‐level variation, with around two‐fold difference between top and bottom 2.5%. Individual preventive services after recall examinations showed the highest variation with over 30‐fold difference between top and bottom 2.5%. All other outcomes showed around 3‐ to 8‐fold differences between practices at top and bottom 2.5% across all examination types. The differences across practices were smaller—1.2‐ to 3.0‐fold—when the top 25% and bottom 25% were compared instead.

**Conclusions:**

This study found considerable variation in diagnostic, preventive, and treatment services provided for Danish adults who underwent a dental examination. The findings highlight the need for research that can inform evidence‐based practice through the development of quality clinical practice guidelines, continuing education programmes, and closer surveillance of care delivery.

## 1 Introduction

In dentistry, as well as in medicine, there is considerable evidence of variations in clinical diagnoses and treatment decisions when identical/similar clinical cases are assessed [1, 2, 3, 4, 5, 6]. Variation in care has also been noted across countries [7, 8] and regions within countries [9, 10] and among healthcare providers in real‐world settings [11, 12]. Timely diagnosis, prevention, and treatment in dentistry are regarded as crucial. However, each method for diagnosis, prevention, or treatment carries potential benefits and harms, necessitating their thoughtful and judicious application under conditions of considerable uncertainty that may stem from limited or poor quality evidence [13] or from diagnostic uncertainty [12]. Both limitations leave room for a substantial provider impact on the care decisions [12]. Provider characteristics such as experience, skills, diligence, tolerance for uncertainty, knowledge and beliefs about treatment utilities, treatment preferences, diagnostic techniques used, outlier experiences, pressures of practice busyness, and adherence to guidelines are some of the factors that have been proposed as influential [11, 14, 15]. While surprisingly little is known about their relative contribution to variation in service provision, the available evidence points to a considerable contribution of provider‐related drivers of the variation in the care provided [15, 16].

These variations represent one pathway through which under‐ or overdiagnosis, and consequently under‐ or overtreatment of dental caries and periodontal conditions can occur, and these issues have been subjects of long‐standing discussions worldwide [17, 18, 19, 20, 21, 22]. Variations among clinical dental practices are also regularly caught and discussed in lay media [23, 24] and have received attention from policymakers and public authorities catering for the quality and equality of the health care system [25]. It is thus clear that if “two dentists consistently provide a different set of preventive and treatment procedures for patients with similar conditions, then one dentist must be providing less effective care than the other, unless the care leads to equivalent results for patients when compared across a wide range of possible outcomes.” [1] However, even if the clinical outcomes may not differ, different sets of preventive and treatment procedures will be associated with different burdens to patients in terms of costs, time commitments, physical and psychological discomfort. These differences also create different burdens for society, particularly when services are subsidised.

In Denmark, dental health care for adults aged 18+ years (22+ years from 2022) is by law organised by the Regional Health Administration and is carried out by private dental practitioners, with whom the Regional Health Administration has made a contract [26]. This contract provides the framework for the provision of those diagnostic, preventive, and therapeutic services for which the Regions provide subsidies. These services include clinical diagnostic procedures, bitewing radiographs, preventive procedures, as well as restorative procedures (fillings, periodontal treatment, endodontics, tooth extractions and surgical removal of teeth), whereas the rehabilitation services (crowns, bridges, implants, partial and full dentures) are not publicly subsidised. Dentists are allowed to contact their patients for recall purposes. Citizens who belong to special needs subgroups whose dental care cannot be undertaken by private dental practitioners are cared for through dental services established by the Danish municipalities. These subgroups include frail elderly, disabled persons, and homeless people, all of whom experience difficulties in making use of the private practice services.

Since 2015, the dentists working in the Danish adult oral health care system have been required to use one of three types of clinical examinations as the basis for their assessment of the optimal recall interval for patients [27]. The extended diagnostic examination is a first‐time examination given to new patients presenting with substantial disease activity and complex treatment need, or to regular patients with sudden manifest disease activity owing to special general or local health conditions. The basic diagnostic examination is also a first‐time examination, given to new patients who do not present with the conditions warranting the extended diagnostic examination. The contents of the extended and basic diagnostic examinations are the same, comprising anamnesis taking, examination, recording observations, diagnosis, treatment planning, risk evaluation, determination of recall interval, and general prevention (information), but the fee for the former is about twice that of the latter. The status examination (recall examination) is given to regular patients participating in the recall system and entails an update of the previous assessment (anamnesis, examination, record taking, diagnosis, treatment planning, risk evaluation, determination of recall interval, and general prevention). The fee for this recall examination is the same as for the basic diagnostic examination.

The actual treatments that follow diagnosis and treatment planning may include disease intervening services, as well as rehabilitation services. While the former services (restorative treatments, endodontics, periodontal treatments, tooth extractions, and individual prevention) are publicly subsidised and therefore tallied in the National Health Insurance Register, rehabilitation services (crowns, bridges, implants, dentures, and splints) are not subsidised and therefore not recorded in the register. While it is plausible that much variation in the oral health care service provision for adults may relate to the rehabilitation aspects, it is nonetheless of great interest to explore the variation in the disease intervening services [28]. The first step on the road to improved quality of care is thus a mapping of the extent to which similar patients receive different care.

It is therefore of both scientific and public health interest to examine the practice‐level differences in the use of common diagnostic, preventive, and treatment procedures. Such analyses are fundamental for attempts to drive improvements in quality, e.g., through education and regulation, eventually benefiting both patients and society in Denmark. As an example, the Regional Health Administrations attempt to control practice‐level variation through socalled control‐statistics. Practices that deviate from the national average by 25% or more may be required to account for their use of specific services, a maximum may be set or a refund may be requested by the Danish regions. However, those data are not publicly available, nor are they available to all relevant stakeholders, and we have therefore found it of interest to present national statistics on the practice‐level variation in common dental diagnostic, preventive, and care services provided for Danish adults who underwent a dental examination during the first 9 months of 2019.

## 2 Material and Methods

The data used for the present analysis originates from a large Danish register‐based dynamic cohort study. The data included all permanent residents of Denmark aged 20 years or older from January 1, 1990, to December 31, 2021. Individuals entered the cohort in the year they reached the age of 20 years and were censored upon death or emigration. Via pseudonymized individual identification numbers assigned to all residents of Denmark in the Civil Registration System, information from the Educational Register, the Income Statistics Register, the National Health Insurance Service Register, and the Register for Selected Chronic Diseases was linked. Dental practices were identified using the provider identification code issued by the Regional Health Administration responsible for organising health care and dental care for the citizens. The individual dentist delivering the service in partnership practices cannot be identified in the National Health Insurance Service Register.

Register‐based studies do not require individual informed consent in Denmark, and the study was approved by the Danish Data Protection Agency.

The study complied with STrengthening the Reporting of OBservational studies in Epidemiology (STROBE) and the Reporting of studies Conducted using Observational Routinely‐collected health Data (RECORD) checklists.

### 2.1 Outcome Window

The National Health Insurance Service Register is an administrative register containing information on all patients, providers, and all subsidised treatment codes in Denmark. First, utilising 2019 data, all patients in dentist practices (specialisation code 50) were identified who had received either (1) an extended diagnostic examination (code 1111), indicated for new patients with high disease activity or complex treatment needs, or if high disease activity or complex treatment needs is caused by sudden manifestation of general disease; (2) a basic diagnostic examination (codes 1112 and 1113) indicated for new patients without high disease activity or complex treatment needs; (3) a status examination (codes 1114 and 1115) indicated for updating the status and planning of necessary preventive and therapeutic interventions.

**FIGURE 1:**
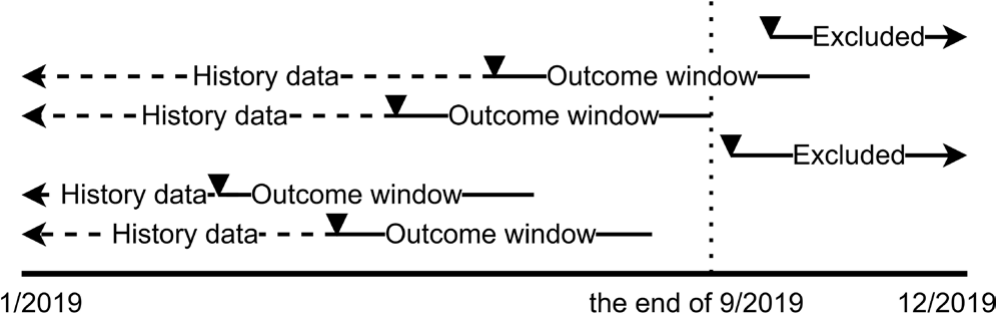
Outcome definition window. The figure illustrates the 2019 dental examination course for six hypothetical patients. Patients were included in the analysis if they had received one of the three relevant types of dental examinations (marked with black triangles) in the period between the first week of 2019 and the last week of September 2019. From this time point, the 13‐week outcome window was defined as the period during which the number of services provided were counted. The hatched lines labelled “history data” indicate that the covariate data used for the case‐mix adjustments were obtained from the registers going back 10 years. The figure also shows that patients who had their first relevant examination in 2019 after the last week of September were excluded as it was not possible to define a 13‐week outcome window for these patients.

The register lacks specific dates for the treatment delivery but contains information about the week when providers invoiced the regions for the delivery of the subsidised treatments. Most of these invoices are registered on the last week of the month. Accordingly, it is reasonable to assume that the services listed in the invoice were delivered during that week or the three preceding weeks of the month. Thus, separately for each of the three types of examinations, the first week of 2019 when the patient received the examination was identified, and then they were followed up for an additional 13 weeks to detect the treatments delivered to the patient by the same provider during this approximately 3 to 4‐month period. This timeframe for the outcome window was selected to allow sufficient time for providers to address all identified treatment needs from the examinations. To maintain equal length of the outcome window after all examinations, only examinations which took place before the end of September 2019 were included (Figure 1).

While an individual could thus appear in the dataset for one or more of the three examination types (e.g., when changing provider or in case of sudden manifest disease activity), only the first examination of each type was considered in the analyses.

### 2.2 Outcomes

Using the data on subsidised dental services delivered during the outcome window period, five binary outcomes reflecting the provision of diagnostic, preventive, and treatment services were formed: (1) supragingival care (including scaling and polishing); (2) subgingival care (excluding periodontal surgery); (3) individual prevention (including oral hygiene advice, fluoride application, smoking, or dietary advice); (4) bitewing radiographs; and (5) endodontic treatment. Additionally, two count outcomes were examined: the number of direct restorations (amalgam or composite) and the number of extractions (surgical and nonsurgical).

### 2.3 Covariates

To take account the possibilities of differences among patients in their actual treatment needs (differing case‐mix) across practices, individual‐level covariates representing sociodemographic characteristics as well as the dental treatments received from the beginning of 2009 to the beginning of the outcome window in 2019 were used to adjust for possible case‐mix differences between practices. Based on the 2019 information, the following covariates were used for these adjustments: age, gender, origin (Danish/immigrant/descendant), region or municipality of residence (15 levels), the highest educational attainment (9 levels), income percentile, and diabetes type 1 or type 2 status (yes/no) (for further detail, see Data S1). Moreover, utilising data from 2009 up to the individual outcome window in 2019, the following variables were formed and used in the case‐mix adjustments: the number of calendar years with at least one dental visit including (1) dental examination, (2) supragingival care, (3) subgingival care, (4) periodontal surgery, (5) individual preventive service, (6) endodontic treatment, or (7) bitewing radiographs. Similarly, the total number of direct restorations and the number of surgical or nonsurgical extractions performed in the period from 2009 up to the individual outcome window in 2019 were calculated and used for the case‐mix adjustment. Additionally, to capture time‐varying and accumulating effects of income, a variable representing the sum of the annual income percentiles between 2009 and 2018 was generated (for further detail, see Data S1).

### 2.4 Sample Selection

**FIGURE 2:**
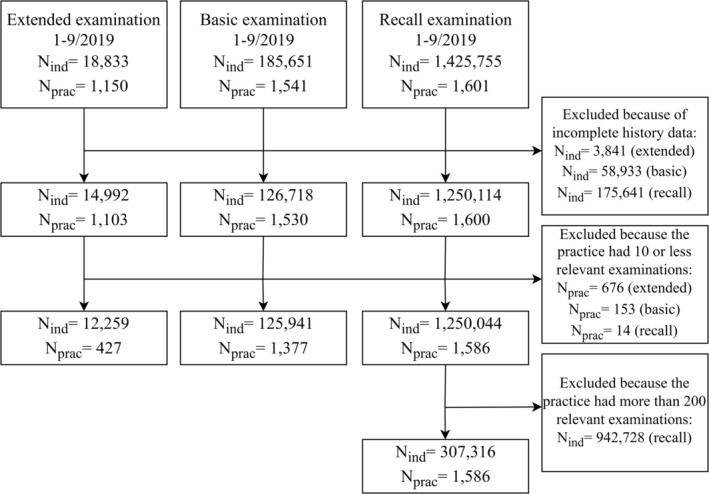
Sample selection process.

The three final analytic samples, representing each distinct type of dental examinations, were generated by omitting data from individuals who had gaps or incomplete covariate information due to residence outside of Denmark or being under 20 years of age for some part of the period 2009–2019. Data were also omitted for practices that conducted 10 or fewer of the relevant types of examinations to ensure more stable and reliable practice‐level estimates. Moreover, to ensure that statistical analyses remained computationally manageable while preserving their validity and generalisability, random samples of 200 status examinations were taken from practices with more than 200 of such examinations. Figure 2 shows the number of practices and individuals that were omitted from analysis for each of these three reasons.

### 2.5 Statistical Analyses

Multilevel Bayesian regression models with weak default priors were used to investigate practice‐level variation in the outcomes. For binary outcomes, analysis was based on logistic regression, while count outcomes were analysed using Poisson regression. First, models were fitted with a random effect for practice but without any individual‐level covariates to capture the unadjusted practice‐level variation in the outcomes. Then, models were fitted which included the random effect for practice and all individual‐level covariates as fixed effects. In these models, the variables of age, income percentile, the sum of income percentiles over 2009–2018, and the total number of direct restorations and the number of surgical or nonsurgical extractions were allowed to have a nonlinear relationship using natural cubic splines with four knots.

Using the outputs from these regression models, practice‐level variation was analysed by computing the practice‐level average predicted outcome probability or count, while keeping individual covariates at their mean or modal values to ensure adjustment for different practice‐level case‐mix. In alignment with data protection rules of the Denmark Statistics, to prevent the identification of individual practices, practice‐level predictions were visualised after being grouped into percentiles.

This investigation also involved reporting the median, the quartile values (25% and 75% percentiles) and the top 2.5% and bottom 2.5% of these practice‐level average predictions. The ratio between the top 2.5% and bottom 2.5% of these practice‐level average predictions was used to express the relative differences between practices with the highest and lowest average predicted outcome probabilities or counts, while excluding the most extreme 2.5% at either end [29]. Since the Danish regions exert their control statistics based on deviations in excess of 25% from the national average, calculations were also done using the ratio between the quartile values (Table S1).

The analyses were performed in R using mainly tidyverse [30], brms [31] and marginaleffects [32] packages on Statistics Denmark's Research Service environment.

## 3 Results

Around 1.5 million adults underwent at least one of the included three types of examinations in around 1600 dental practices between January and September 2019 (Figure 2). After applying the selection criteria for the sample, the final groups consisted of 12 259 individuals who received an extended examination across 427 practices; 125 941 individuals who underwent a basic examination across 1377 practices; and 307 316 individuals who attended a recall examination across 1586 practices (Table 1).

**TABLE 1:** Characteristics of the final analytic samples for the three types of dental examinations.

	Extended	Basic	Recall
*N* (individuals)	12 259	125 941	307 316
*N* (practices)	427	1377	1586
Age, median (Q1, Q3)	55 (44, 67)	51 (40, 64)	59 (47, 70)
Men	6194 (51%)	57 720 (46%)	140 226 (46%)
Origin			
Danish	10 794 (88%)	113 667 (90%)	291 533 (95%)
Immigrant	1348 (11%)	11 003 (8.7%)	14 345 (4.7%)
Descendant	117 (1.0%)	1271 (1.0%)	1438 (0.5%)
Highest educational attainment			
Primary school	3791 (31%)	24 153 (19%)	54 235 (18%)
Vocational education	4610 (38%)	44 867 (36%)	113 977 (37%)
Short‐cycle higher education	504 (4.1%)	6870 (5.5%)	17 198 (5.6%)
Medium‐cycle higher education	1619 (13%)	23 449 (19%)	60 773 (20%)
Long‐cycle higher education	623 (5.1%)	15 297 (12%)	36 768 (12%)
Other (4 levels)	1112 (9.1%)	11 305 (9.0%)	24 365 (7.9%)
Municipality/region			
Central Jutland	1503 (12%)	14 585 (12%)	37 730 (12%)
Southern Denmark	1690 (14%)	13 514 (11%)	33 506 (11%)
Copenhagen	1093 (8.9%)	13 882 (11%)	28 987 (9.4%)
Capital region (outside Copenhagen)	2622 (21%)	27 357 (22%)	76 710 (25%)
Zealand	2030 (17%)	19 361 (15%)	46 188 (15%)
Other region or municipality (10 levels)	3321 (27%)	37 242 (30%)	84 195 (27%)
Income percentile, median (Q1, Q3)	45 (28, 70)	60 (36, 80)	61 (36, 83)
Diabetes	1186 (9.7%)	6952 (5.5%)	17 363 (5.6%)
Sum of income percentiles^a^, median (Q1, Q3)	460 (296, 663)	556 (361, 747)	616 (400, 799)
Sum of restorations^a^, median (Q1, Q3)	4 (1, 10)	4 (1, 8)	6 (3, 11)
Sum of extractions^a^, median (Q1, Q3)	1 (0, 2)	0 (0, 1)	0 (0, 1)
Sum of years with endodontic treatment^a^, median (Q1, Q3)	0 (0, 1)	0 (0, 1)	0 (0, 1)
Sum of years with individual prevention^a^, median (Q1, Q3)	1 (0, 2)	1 (0, 2)	2 (1, 3)
Sum of years with examination^a^, median (Q1, Q3)	3 (1, 7)	5 (2, 8)	9 (7, 10)
Sum of years with subgingival care^a^, median (Q1, Q3)	0 (0, 2)	0 (0, 1)	0 (0, 3)
Sum of years with periodontal surgery^a^, median (Q1, Q3)	0 (0, 0)	0 (0, 0)	0 (0, 0)
Sum of years with bitewing radiographs^a^, median (Q1, Q3)	1 (0, 2)	2 (1, 3)	3 (2, 4)
Sum of years with supragingival care^a^, median (Q1, Q3)	1 (0, 5)	4 (1, 7)	7 (4, 9)

^a^ Over 2009–2019; Q1, Q3 = first and third quartile.

Individuals who received recall examinations were, on average, older than individuals who received extended or basic dental examinations (Table 1). Those who received extended examinations were more likely to be immigrants and men who have lower education, lower income, diabetes, and visited dental care less often, and they were likely to have received fewer examinations and less supragingival care during the previous 10 years than those in the basic or recall examination samples. Differences between individuals in the basic examination and recall examination samples were less clear, but it can be concluded that individuals who were included in the recall examination sample were more likely of Danish origin, older, and having received more restorations, examinations, and supragingival care during the previous 10 years than individuals in the basic examination sample.

Around two‐thirds of individuals who underwent an extended or basic examination also had bitewing radiographs taken within the outcome window (approximately 3–4 months), whereas only one‐quarter of recall examination patients had bitewing radiographs taken (Table 2). Regarding all three types of examinations, around one half of the individuals received individual prevention. While those who had an extended examination were more likely to receive subgingival care (32%) than those who had basic (20%) or recall (24%) examinations, they were significantly less likely to receive supragingival care (33%) than those in the basic (68%) or recall (77%) examination samples. The average number of extractions and restorations received during the outcome window was many fold higher in the extended and basic examination samples than in the recall examination sample.

**TABLE 2:** Diagnostic, preventive, and treatments received during the 2019 outcome window by the examination type.

		Extended	Basic	Recall
*N* (patients)		12 259	125 941	307 316
Bitewing radiograph	*N* (%)	7370 (60%)	83 498 (66%)	81 652 (27%)
Individual prevention	*N* (%)	6139 (50%)	63 825 (51%)	134 263 (44%)
Subgingival care	*N* (%)	3956 (32%)	24 925 (20%)	73 816 (24%)
Supragingival care	*N* (%)	4071 (33%)	86 007 (68%)	235 379 (77%)
Endodontic treatment	*N* (%)	1630 (13%)	8029 (6.4%)	5789 (1.9%)
Number of extractions	Mean (SD) Median (Q1, Q3)	1.26 (3.08) 0 (0, 1)	0.33 (1.40) 0 (0, 0)	0.05 (0.34) 0 (0, 0)
Number of restorations	Mean (SD) Median (Q1, Q3)	2.06 (3.32) 1 (0, 3)	1.01 (1.83) 0 (0, 1)	0.48 (1.04) 0 (0, 1)

*Note:* Q1, Q3 = first and third quartile.

**FIGURE 3:**
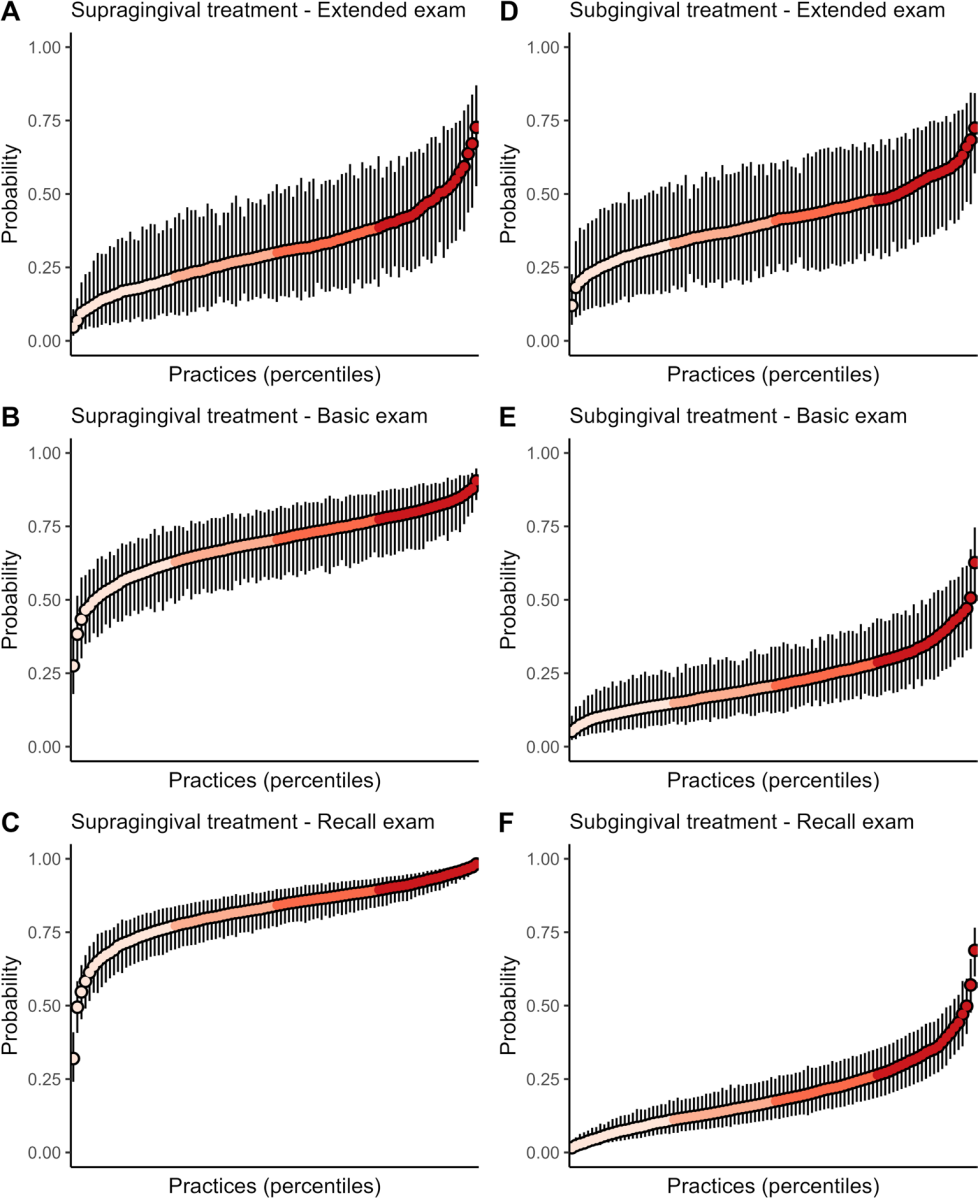
Average predicted probability of supragingival (A–C) and subgingival treatment (D–F) with 95% credibility intervals across practice percentiles for each type of examination. Predictions were generated using multilevel Bayesian regression models, with individual‐level covariates held at their mean or modal values.

**FIGURE 4:**
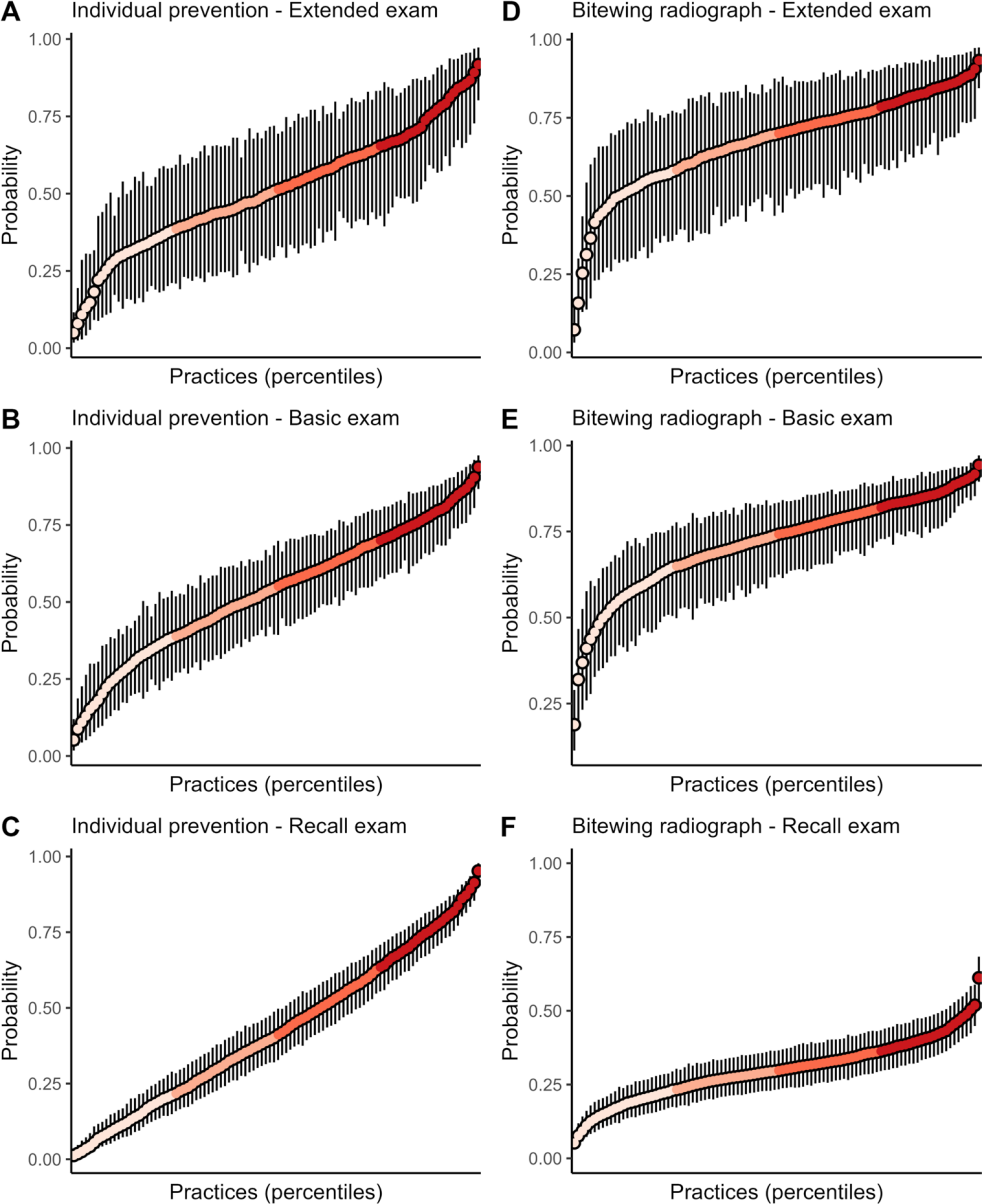
Average predicted probability of individual prevention (A–C) and bitewing radiographs (D–F) with 95% credibility intervals across practice percentiles for each type of examination. Predictions were generated using multilevel Bayesian regression models, with individual‐level covariates held at their mean or modal values.

**FIGURE 5:**
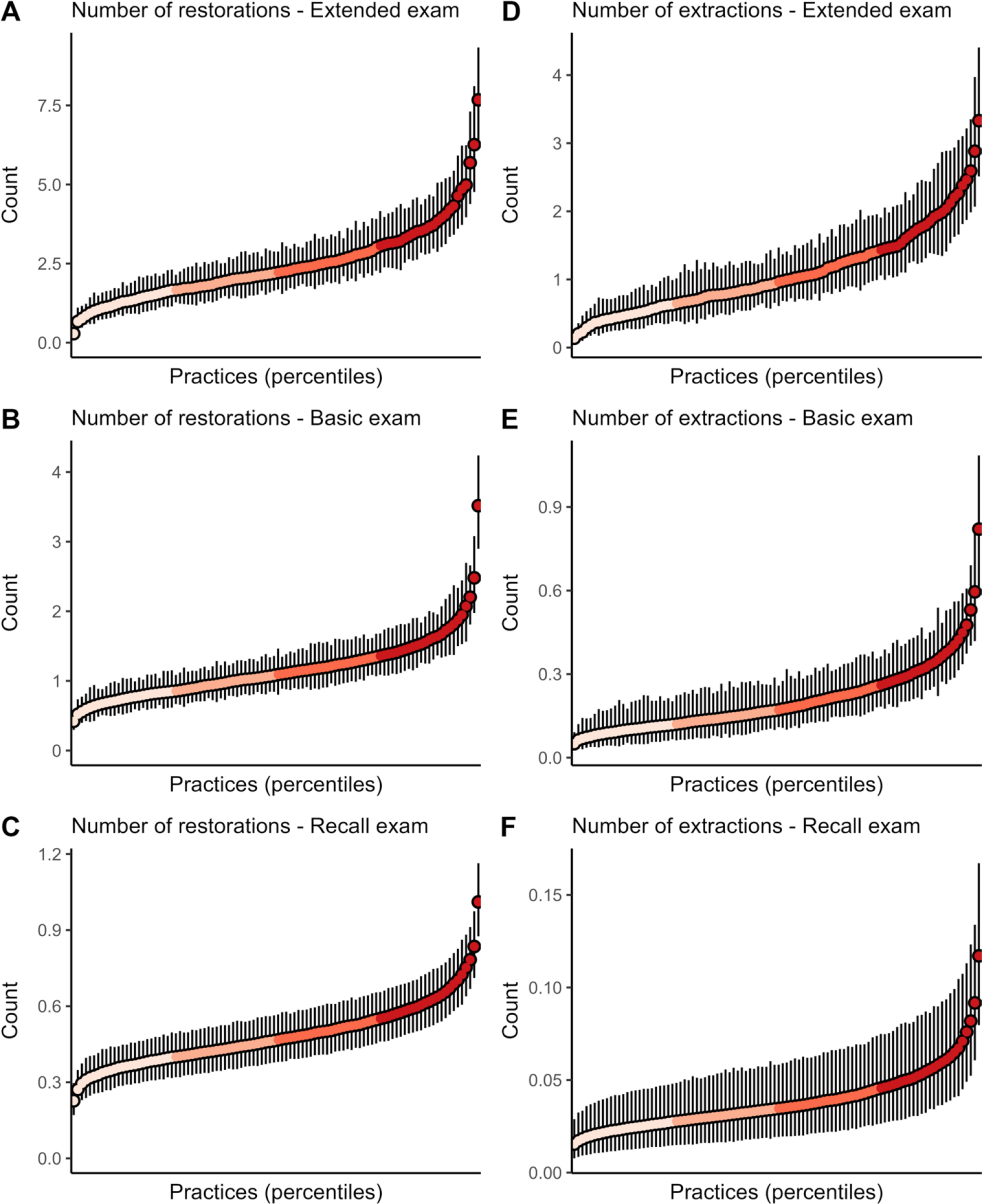
Average predicted count of restorations (A–C) and extractions (D–F) with 95% credibility intervals across practice percentiles for each type of examination. Predictions were generated using multilevel Bayesian regression models, with individual‐level covariates held at their mean or modal values.

The multilevel Bayesian regression analyses indicated that, in all three examination types, considerable variability was observed in the average predicted outcome probabilities or counts between practices (Table 3, Figures 3, 4, 5). Except for the outcome number of extractions, the adjustment for individual‐level covariates did not notably reduce the variation across practices. In the following, the adjusted estimates are therefore quoted. The smallest variation across practices (on the relative ratio scale) occurred in the probability of providing supragingival care after the basic or the recall examination (Figure 3B,C). Following these two examination types, practices in the top 2.5% for predicted probability were approximately twice as likely to provide supragingival care compared to practices in the bottom 2.5%. Practices in the top 25% for the predicted probability were approximately 1.20 times more likely to provide this service than were practices in the bottom 25% (Table S1), as also indicated by the flatness of the central portions of the curves in Figure 3B,C. However, this relatively small variation should be seen in the light of the generally high predicted probability of provision of this service, being 70% following a basic examination and 84% following a recall examination (Table 3, Figure 3B,C). In contrast, the ratio between the top 2.5% and bottom 2.5% in average predicted probability of providing individual preventive service after a recall examination was over 30 (Table 3, Figure 4C). There were also over 10‐fold relative differences across practices in the probability of providing subgingival care after a recall examination (Table 3, Figure 3F), and in the predicted number of extractions performed after an extended examination (Table 3, Figure 5D). Otherwise, ratios between the top 2.5% and bottom 2.5% of these practice‐level average predictions indicated that the top 2.5% had around 3–8 times higher average predicted outcome probability or count than the bottom 2.5% in all examination samples (Figures 3, 4, 5 and S1).

**TABLE 3:** Unadjusted and adjusted average predicted outcome probability or count across practices.

	Extended		Basic		Recall	
	Unadjusted	Adjusted^a^	Unadjusted	Adjusted^a^	Unadjusted	Adjusted^a^
	Median p2.5; p97.5 Ratio^b^	Median p2.5; p97.5 Ratio^b^	Median p2.5; p97.5 Ratio^b^	Median p2.5; p97.5 Ratio^b^	Median p2.5; p97.5 Ratio^b^	Median p2.5; p97.5 Ratio^b^
Bitewing radiograph	0.63 0.23; 0.86 3.78	0.69 0.24; 0.89 3.71	0.66 0.31; 0.87 2.81	0.74 0.37; 0.91 2.44	0.26 0.09; 0.46 5.12	0.29 0.09; 0.50 5.28
Individual prevention	0.50 0.11; 0.87 7.93	0.49 0.10; 0.86 8.30	0.50 0.10; 0.86 8.49	0.54 0.11; 0.88 7.96	0.42 0.02; 0.91 43.13	0.40 0.02; 0.89 35.81
Subgingival care	0.32 0.15; 0.59 4.06	0.39 0.20; 0.65 3.31	0.19 0.08; 0.41 5.03	0.20 0.07; 0.46 6.30	0.22 0.03; 0.56 19.54	0.17 0.03; 0.49 16.82
Supragingival care	0.32 0.12; 0.67 5.62	0.29 0.09; 0.62 6.70	0.72 0.46; 0.87 1.87	0.70 0.43; 0.87 2.00	0.78 0.48; 0.95 2.00	0.84 0.55; 0.97 1.77
Endodontic treatment	0.12 0.06; 0.28 4.93	0.14 0.07; 0.32 4.32	0.05 0.03; 0.13 4.05	0.06 0.04; 0.12 3.31	0.02 0.01; 0.04 3.68	0.02 0.01; 0.03 2.73
Number of extractions	1.07 0.17; 4.18 24.52	0.90 0.22; 2.51 11.58	0.22 0.07; 0.89 12.79	0.17 0.07: 0.52 7.58	0.04 0.02; 0.12 6.06	0.03 0.02; 0.08 4.39
Number of restorations	2.02 0.66; 4.80 7.23	2.13 0.74; 5.34 7.22	0.98 0.47; 2.14 4.51	1.07 0.56; 2.18 3.92	0.45 0.24; 0.91 3.71	0.46 0.30; 0.78 2.64

^a^ Multilevel Bayesian regression models were adjusted for age, gender, origin, municipality/region, the highest educational attainment, income percentile (2019), diabetes status, sum of years with (1) dental examination, (2) supragingival care, (3) subgingival care, (4) periodontal surgery, (5) individual preventive service, (6) endodontic treatment, or (7) bitewing radiographs, the sum of (1) restorations and (2) extractions from 2009 to the individual outcome window, and the sum of annual income percentiles between 2009 and 2018.

^b^ 97.5th percentile (p97.5)/2.5th percentile (p2.5).

## 4 Discussion

This study investigated practice‐level variation in dental services for Danish adults who underwent one of three different dental examination types. Substantial variability was observed across practices in the provision of diagnostic, preventive, and restorative services. Comprehensive case‐mix adjustment for individual‐level covariates (including demographic and socioeconomic factors, as well as 10‐year history of dental treatments) did not considerably alter the variation observed across practices. The variation across practices occurred across all examination types. Overall, the provision of the investigated diagnostic, preventive, and treatment services was around 2‐ to 8‐fold higher in practices in the top 2.5% than in the bottom 2.5%. The highest variation across practices occurred in providing individual prevention (36‐fold) and subgingival care (17‐fold) after a recall examination, and in the number of extractions (12‐fold) following the extended diagnostic examination for patients with high disease activity or complex treatment needs. When comparing the top 25% and bottom 25% instead, the differences across practices were smaller, ranging from 1.2‐ to 3.0‐fold.

The practice‐level variation described here clearly exceeds the ±25% limits for the tolerable variation in the use of services laid down in the contract between the Danish Regional Health Administrations and the Danish Dental Association [26]. In the best interests of patients and society at large, the regions attempt to monitor the quality of care provided through annual control statistics. These control statistics analyse the level of service provision for all services described in the contract, adjusted for patient case‐mix in terms of age and gender. The possible practice status as a referral practice for certain procedures is also taken into account. The findings for each practice are benchmarked against regional and national averages, and undue variation is identified and explored in an attempt to guide practices towards similar care. However, as the case‐mix adjustment is more comprehensive and eligibility criteria more uniform in the present study than that used by the Regional Health Administrations, the findings indicate that sizeable variation may exist despite these administrative efforts. Crucially, neither regional/national averages nor average predicted probabilities/counts detected here can be used alone to judge which are the more correct service provision levels. Such inference would necessitate establishing clinical norms based on long‐term studies, preferably randomised clinical trials, investigating the relationship of the care provided or diagnostic services used with the patient‐important outcomes, such as tooth retention, and costs. This information could then be used to develop nationally endorsed clinical practice guidelines, evidence‐based continuing education programmes, and closer surveillance of care delivery and diagnostic practices, to guide practices towards more optimal care and diagnostic practices. However, what a study such as the present can tell is that dental practices vary considerably in their treatment norms for apparently similar patients, which is generally considered undesirable. This is also reflected by the administrative, educational, and guideline efforts aimed at standardising the care of similar patients. These efforts are consistent with the broader objective of achieving horizontal equity (equal treatment for equal need) within healthcare systems [33].

Clearly, some of the variation in service provision could be explained by some practices offering services as referral practices for e.g., periodontal therapy or endodontics. In Denmark, only two specialisations exist (orthodontics and oral and maxillofacial surgery), and the referral practices are therefore typically clinics in which one or more of the associated providers have acquired special competences within the area of referral. Such practices will necessarily be characterised by higher levels of service provision within their respective expertises. These practices are not officially registered, and it is therefore not possible to exempt them from the analysis. However, as they are few, they are unlikely to substantially affect the analyses. Hence, as of late 2024, 22 Danish practices offered referral services for endodontics, while 11 offered referral services for periodontal therapy [34].

It was not possible to account for patient preferences and values possibly influencing the service provision. However, patient values and preferences would be an important and acceptable, not to say preferable, source of variation across practices. Previous studies have shown that dentists tend to be somewhat hesitant to implement shared decision‐making in a clinical context [35, 36, 37, 38], and it is not known to what extent care provision is really shaped by patient preferences and values. Moreover, it is likely that patient views are weighed differently for diagnostic, preventive, or more invasive procedures (like extractions), making it challenging to speculate how high the variation across practice would have been had it been possible to adjust for patient preferences and values. Presumably, there would still have been considerable variation in clinical practice across providers.

Similarly, because studies on identical cases [1, 2, 3, 4, 5, 6] have shown that there exists considerable variation in diagnostic and treatment practices among professionals, it is likely that there would still have been considerable variation across practices, even if it had been possible to adjust more accurately for patient dental and general health status. Accordingly, the comprehensive adjustment set employed here did not considerably explain the variation across practices.

Other limitations of the study that deserve mention include some uncertainties regarding the timing of services. Due to not having the actual dates of treatment delivery in the register, there may be some uncertainty about the temporal sequence of examinations and services provided within the month of examination. Hence, reimbursement claims are usually sent to the Danish regions at the end of each month, and it is therefore possible, but unlikely on a large scale, that some of the services have actually been provided before the examination. In any case, they are still conducted by the same practice. The register data also lacks individual practitioner‐level information, since clinics/practices are identified by a provider number that covers all dentists working in that practice. This number may vary from a single dentist to several professionals (dentists and dental hygienists), all working under one and the same provider number. It was therefore not possible to identify the individual professionals or their profession, which may also explain some of the variation observed, e.g., in the recall examinations which can be conducted by either dentists or dental hygienists depending on the practice. Considering the bigger dental practices, it is likely that considerable within practice variation may exist, which could thus not be captured or controlled here. Even though the generalisability of these findings to other countries may be limited (e.g., due to different treatment codes and health care systems), it is highly probable that quite similar variations in dental practices occur globally.

Despite these limitations, it is reasonable to discuss the implications of the observed level of service provision and to consider the factors underlying practice‐level variation. The observed practice‐level variation must also necessarily be seen in the light of the level of service provision. Whereas supra‐ and subgingival care was almost equally likely after an extended examination, as indicated by the average predicted probabilities of 29% and 39% (Table 3, Figure 3A,D), respectively, supragingival care was much more likely than subgingival care after a basic examination (70% vs. 20%, Figure 3B,E), and this contrast was even accentuated after the recall examination (84% vs. 18%, Figure 3C,F). Considering that service provision was generally lower after a recall examination than after the other two examination types, this indicates that the provision of supragingival treatment to 84% of the recall patients might represent a ‘sales‐compensating’ act, i.e., the provision of this service to compensate for the lack of other chargeable procedures. This interpretation would align with previous suggestions that fee‐for‐service dentist may have set a minimum number of procedures per patient for their work to be worthwhile in financial terms [39]. It is hardly likely that recall patients should be more plagued by mineralized plaque and calculus or stains than either type of new patients receiving an extended examination or a basic examination. The relatively limited variation observed in the provision of supragingival therapy following both the recall examination and the basic examination further indicates that the dental practices generally agree on a practice involving a high frequency of provision of this service. A Cochrane review has shown that routine scaling and polishing reduces calculus more than no routine scaling and polishing, and that a 6‐monthly routine works better than a 12‐monthly routine in calculus reduction [40]. However, there is high‐certainty evidence that the routine scale and polish treatment makes little or no difference to gingivitis, probing depths and oral health‐related quality of life [40]. This is consistent with evidence that restricting the frequency of supragingival treatment to no more than every second year, resulted in practically the same 10‐year cumulative incidence of tooth loss as under the observed periodontal care pattern in Denmark [41].

The provision of bitewing radiographs following a dental examination varied considerably across practices. While some practices had taken radiographs from less than 50% of their extended or basic examination patients and less than 10% of their recall examination patients, other practices had taken radiographs from practically all their extended or basic examination patients and 50% of their recall examination patients. These findings are somewhat surprising because bitewing radiographs work best in detecting cavities when sufficiently deep [42, 43, 44], which we would expect to see more often in patients requiring extended examinations instead of basic examinations. Whereas the number of untreated and cavitated caries lesions may generally be expected to be quite low in recall patients (of those, 29% received restorative treatments, 0.48 treatments on average, Table 2), it is quite interesting that some practices used bitewing radiographs for their recall patients as frequently as some other practices used them for patients undergoing extended or basic examinations. This may, of course, be explained by radiographic follow‐up of non‐operatively treated dentin caries lesions or an unwarranted, but perhaps surprisingly common, belief that bitewing radiographs would detect early lesions [42, 43, 44]. However, in our view, it is likely that high bitewing radiograph use among recall patients, at least in some practices, may entail a risk of overdiagnosis and therefore unnecessary restorative intervention [45] and radiation exposure.

In view of the indications for the different types of examinations, it was remarkable that the level was quite uniform and the variation pronounced regarding the provision of individual prevention services across examination types. Individual prevention service provision probabilities ranging between 0 and 1 for all examination types, and the average probabilities were 0.49, 0.54 and 0.40 for the extended, the basic and the recall examination, respectively. One might have expected a substantially higher level of provision of this service and less variation following the extended examination which is indicated for new patients presenting with substantial disease activity and complex treatment need, or regular patients with sudden manifest disease activity owing to special general or local health conditions. The level of and variation in the provision of the individual preventive services after recall examinations was also surprising, given that the majority of these patients had been examined annually, or almost annually, during the last 10 years (median: 9 years, Q1: 7 years, Q3: 10 years; Table 1). According to legal regulations, individual preventive services should be provided for patients with active caries, gingivitis, periodontitis and peri‐implantitis, or other dental disease requiring preventive treatment. Unfortunately, the meaning of ‘active’ oral disease is not defined in the regulation, and both the level and the variation in service provision may therefore indicate considerable disagreements regarding the indications for the individual preventive service. If, for instance, any sign of gingival inflammation [46] or modest clinical attachment loss [47] is taken to indicate a need for the individual preventive service, e.g., to prevent periodontal attachment loss, it is clearly possible to provide this service to essentially all recall patients in Denmark [48] and elsewhere [49, 50]. While this would be a gross exaggeration of the importance of limited bleeding on probing or modest clinical attachment loss for maintaining oral health, these positions clearly indicate how different disease concepts may influence provision [51, 52]. In any case, it remains to be seen if active oral disease is as prevalent as indicated by the use of the individual preventive service among users of the Danish oral health care services.

The current study found considerable variation in diagnostic, preventive, and care services provided for Danish adults who underwent a dental examination. The findings highlight the need for research that can inform evidence‐based practice through the development of quality clinical practice guidelines, continuing education programs, and closer surveillance of care delivery. It is also essential that patients, professionals and regulators are kept informed about the extent and scale of these variations at the local level, as well as their trends over time.

## Author Contributions

**Eero Raittio:** conceptualisation; formal analysis; methodology; investigation; visualisation; writing – original draft; and writing – review and editing. **Vibeke Baelum:** conceptualisation; data curation; investigation; project administration; supervision; writing – original draft; and writing – review and editing.

## Ethics Statement

The study was approved by the Danish Data Protection Agency (2015‐57‐0002) and Aarhus University (2016‐051‐000001‐914).

## Consent

The authors have nothing to report.

## Conflicts of Interest

The authors declare no conflicts of interest.

## Supporting information


Data S1.
**Table S1.** Unadjusted and adjusted average predicted outcome probability or count across practices.
**Figure S1.** Average predicted probability of endodontic treatment with 95% credibility intervals across practice percentiles for each type of examination (A–C). Predictions were generated using multilevel Bayesian regression models, with individual‐level covariates held at their at their mean or modal values.

## Data Availability

The data that support the findings of this study are available from Statistics Denmark. Restrictions apply to the availability of these data, which were used under licence for this study.
