# Supplementary material for: Practice‐Level Variation in the Provision of Subsidised Dental Services to Adult Danes in 2019: A Register‐Based Study
Source: Community Dent Oral Epidemiol. 2025 Jun 3;53(4):452–64. doi: 10.1111/cdoe.13048 (PMC12238737; doi:10.1111/cdoe.13048)
Supplement: Supplementary file 1 — Data S1. Table S1. Unadjusted and adjusted average predicted outcome probability or count across practices. Figure S1. Average predicted probability of endodontic treatment with 95% credibility intervals across practice percentiles for each type of examination (A–C). Predictions were generated using multilevel Bayesian regression models, with individual‐level covariates held at their at their mean or modal values. [file CDOE-53-452-s001.docx]

**Supplementary information**

The following registers were used to build the research database, using the fact that individual level data can be linked across registers using the Civil Registration Number as the key:

The Danish Civil Registration System. Regarding validity and coverage, it is generally accepted that the information recorded is of very high quality. (Pedersen 2011)

The Danish Educational Register. Regarding coverage, 97% of the Danish population aged 15-69 had non-missing education information, and for the immigrant population born in the same cohorts, the coverage was 85-90%. The register information is considered of high validity although variations are seen across different populations and cohorts (Jensen and Rasmussen 2011, Pallesen et al 2010)

The Danish Income Statistics Register. Regarding validity and coverage the information in this register is considered to be of high quality, particularly when using income definitions of relevance to the administrative authorities (Baadsgaard and Quitzau 2011)

The National Health Insurance Service Register. Regarding coverage, it is generally accepted that a strength of this register is its completeness, size, and follow-up period. Regarding accuracy it must be noted that there is an economic incentive for providers to overreport their services. However, the providers have to explain their invoices to the Regional Health Adminstration if the number of services provided per patient exceed 25% of the average for providers in the region (Andersen et al. 2011).

The Registry for Selected Chronic Diseases and Severe Mental Disorders. This register combines information from the Danish National Prescription Registry (Pottegård et al. 2017), which covers all prescriptions in Denmark, and the Danish National Patient Registry (Schmidt et al. 2015) which contains information on all visits, procedures, and admissions to all Danish somatic hospitals, emergency departments, and hospital-associated outpatient clinics. Some underestimations may be expected if patients are treated outside hospitals and either do not receive prescription medicine or receive medicine that can be used for other purposes. While the algorithms for defining a person as a diabetic are not carved in stone, completeness is considered high, although tweaks of the defining algorithms have been suggested to reduce the possibility of misclassification (Carstensen & Jørgensen 2018). A validation study (Isaksen et al. 2023) has shown that type II diabetes identification method in the Register for Selected Chronic Diseases has sensitivity of 0.91 (95% confidence interval, CI: 0.89-0.92) and positive predictive value of 0.90 (95% CI: 0.88-0.91) when compared to self-reported type II diabetes status. In the case of type 1 diabetes, sensitivity 0.70 (95% CI: 0.65-0.74) and positive predictive value 0.94 (95% CI: 0.91-0.97) were slightly lower.

**Variables**

**Gender**: Men; Women. Information from: The Danish Civil Registration System (Pedersen 2011). Variable name in the register: KOEN.

**Origin**: Categorized into two groups: 1) Immigrants or descendants, and 2) persons of Danish origin. (Pedersen 2011). Variable name in the register: IE_TYPE

**Region/Municipality of residence**: Municipality if a person lived in one of ten biggest municipalities (Copenhagen, Aarhus, Odense, Aalborg, Vejle, Kolding, Esbjerg, Viborg, Randers, Frederiksberg) and one of the regions otherwise. Information from: The Danish Civil Registration System (Pedersen 2011). Variable names in the register: KOM and REG.

**Highest completed education**: categorized into eight groups:

- Primary school
- High school
- Vocational
- Short-cycle higher education or qualifying exam
- Medium-cycle higher education
- Bachelor
- Long cycle higher education
- Researcher education
- Unknown

Information from the Danish Educational Register (Jensen and Rasmussen 2011, Pallesen et al 2010). Categorized based on the variable in the register AUDD. Qualifying exam and Researcher education categories were combined with closest neighboring category due to their small size.

**Income percentile**: People were divided into 100 groups (percentiles) for each year from 2009 to 2019 based on their total annual personal income. Personal income in total is equal to the sum of business income, transfer income, property income (excluding calculated rental value of own home) and other non-classifiable income that can be attributed directly to the individual. The amount is before tax deduction, labor market contribution and special pension contribution, and interest expenses are not deducted. Information from the Danish Income Statistics Register (Baadsgaard and Quitzau 2011). Variable name in the register: PERINDKIALT_13.

**Dental service variables**

From the National Health Insurance Service Register (Andersen et al. 2011), we used the dental treatment codes that have been used over time since 2009 in the National Health Insurance scheme covering the subsidized dental care for all adult permanent residents in Denmark. According to the descriptions of the codes, services were categorized as follows:

| **Service** | **Codes** | **Details** |
| --- | --- | --- |
| Supragingival treatment | 1301, 1302 | Supragingival periodontal treatment (scale and polish) |
| Subgingival treatment | 1420, 1425, 1430, 1452, 1453, 1431 | Subgingival periodontal treatment (instrumentation) and control |
| Periodontal surgery | 1440, 1454 | Periodontal surgery and control |
| Dental restorations | 1501, 1502, 1503, 1504, 1505, 1506, 1507, 1509, 1551, 1552, 1553, 1554, 1555, 1556, 1557, 1558, 1559 | Amalgam and composite restorations |
| Oral examination | 1111, 1112, 1113, 1114, 1115, 1116, 1140, 1141, 1160, 1170, 1171, 1180, 2910, 1415 | Focused (e.g., periodontal) or general examinations and dental check-ups, includes also general oral hygiene advice |
| Individual prevention | 2920, 2930 | Individualized oral hygiene advice, fluoride application, smoking advice, or dietary advice |
| Bitewing radiograph | 1150, 1151, 1152, 1153 | - |
| Tooth extractions | 1701, 1705, 1801 | Non-surgical extractions and surgical extractions (i.e. with gingival or mucosal incision, root sectioning or removal of bone tissue) |
| Endodontic treatment | 1600, 1601, 1605, 1606 | Root canal treatments, periapical surgery, and pulpotomies |

**Incident diabetes mellitus type 1 or 2:** Data on incident diabetes between 1997 and 2019 comes from the Registry for Selected Chronic Diseases and Severe Mental Disorders (The Danish Health Data Authority 2024).

Individuals were classified as having incident diabetes mellitus (type 1 or 2) in the beginning of 2019 if the person meet one of the following criteria between 1997 and 2018.

Diabetes type 1:

- Individuals registered with at least two purchases of insulin or insulin analogs (A10A, except combination medicines including GLP1-analogues and insulins, A10AE54 or A10AE56) in the Danish National Prescription Registry.
- Individuals registered with a relevant primary or secondary diagnosis (E10, diabetes type I, or its sub-codes under ICD-10) in the Danish National Patient Registry.

Diabetes type 2:

- Individuals registered with at least two purchases of medication aimed at lowering blood glucose (A10B, except A10BJ, A10BK01, A10BK03) or combination medicines including GLP1-analogues and insulins (A10AE54 or A10AE56) in the Danish National Prescription Registry.
- Individuals registered with a relevant primary or secondary diagnosis (E11, diabetes type 2, or its sub-codes under ICD-10) in the Danish National Patient Registry.

Excluded were:
Women who have been exclusively treated with metformin (ATC code A10BA02) and there were signs that they could have polycystic ovary syndrome (prescription for G03GB02, G03HB or diagnosis code E282).

Women who have a code for gestational diabetes (ICD-10 code O24.4) and who have only registered purchase of antidiabetics (A10) within 280 days before first contact or 280 days after last contact with gestational diabetes according to the Danish National Patient Registry

**References**

Andersen JS, Olivarius Nde F, Krasnik A. The Danish National Health Service Register. Scand J Public Health. 2011;39(7 Suppl):34-7

Baadsgaard M, Quitzau J. Danish registers on personal income and transfer payments. Scand J Public Health. 2011;39(7 Suppl):103-5.

Carstensen B, Jørgensen ME. Danish Diabetes Registers: RUKS & DMreg. https://bendixcarstensen.com/DMreg/RUKScmp.pdf

The Danish Health Data Authority. Registry for Selected Chronic Diseases and Severe Mental Disorders [in Danish]. 2023. https://www.esundhed.dk/Emner/Operationer-og-diagnoser/Udvalgte-kroniske-sygdomme-og-svaere-psykiske-lidelser

Isaksen AA, Sandbæk A, Bjerg L. Validation of Register-Based Diabetes Classifiers in Danish Data. Clin Epidemiol. 2023 May 5;15:569-581.

Jensen VM, Rasmussen AW. Danish Education Registers. Scand J Public Health. 2011;39(7 Suppl):91-4.

Pallesen PB, Tverborgvik T, Rasmussen HB, Lynge E. Data on education: from population statistics to epidemiological research. Scand J Public Health. 2010;38(2):177-83.

Pedersen CB. The Danish Civil Registration System. Scand J Public Health. 2011;39(7 Suppl):22-5.

Pottegård A, Schmidt SAJ, Wallach-Kildemoes H, Sørensen HT, Hallas J, Schmidt M. Data Resource Profile: The Danish National Prescription Registry. Int J Epidemiol. 2017 Jun 1;46(3):798-798f.

Schmidt M, Schmidt SA, Sandegaard JL, Ehrenstein V, Pedersen L, Sørensen HT. The Danish National Patient Registry: a review of content, data quality, and research potential. Clin Epidemiol. 2015 Nov 17;7:449-90.

Table S1. Unadjusted and adjusted average predicted outcome probability or count across practices.

|  | **Extended** | | **Basic** | | **Recall** | |
| --- | --- | --- | --- | --- | --- | --- |
|  | Unadjusted | Adjusted^1^ | Unadjusted | Adjusted^1^ | Unadjusted | Adjusted^1^ |
|  | Median  Q1; Q3  Ratio^2^ | Median  Q1; Q3  Ratio^2^ | Median  Q1; Q3  Ratio^2^ | Median  Q1; Q3  Ratio^2^ | Median  Q1; Q3  Ratio^2^ | Median  Q1; Q3  Ratio^2^ |
| Bitewing radiograph | 0.63  0.52; 0.72  1.40 | 0.69  0.56; 0.77  1.37 | 0.66  0.56; 0.75  1.34 | 0.74  0.65; 0.81  1.26 | 0.26  0.20; 0.32  1.57 | 0.29  0.23; 0.36  1.55 |
| Individual prevention | 0.50  0.37; 0.65  1.75 | 0.49  0.36; 0.64  1.78 | 0.50  0.35; 0.66  1.91 | 0.54  0.38; 0.69  1.80 | 0.42  0.22; 0.65  3.00 | 0.40  0.21; 0.62  2.92 |
| Subgingival care | 0.32  0.24; 0.40  1.62 | 0.39  0.32; 0.48  1.51 | 0.19  0.14; 0.25  1.81 | 0.20  0.15; 0.28  1.90 | 0.22  0.15; 0.31  2.09 | 0.17  0.11; 0.26  2.35 |
| Supragingival care | 0.32  0.22; 0.42  1.90 | 0.29  0.20; 0.38  1.91 | 0.72  0.64; 0.78  1.22 | 0.70  0.63; 0.77  1.23 | 0.78  0.70; 0.85  1.21 | 0.84  0.77; 0.89  1.16 |
| Endodontic treatment | 0.12  0.09; 0.16  1.69 | 0.14  0.11; 0.18  1.60 | 0.05  0.04; 0.07  1.60 | 0.06  0.05; 0.07  1.49 | 0.02  0.01; 0.02  1.54 | 0.02  0.01; 0.02  1.46 |
| Number of extractions | 1.07  0.60; 1.81  3.03 | 0.90  0.60; 1.39  2.31 | 0.22  0.13; 0.37  2.77 | 0.17  0.12: 0.25  2.15 | 0.04  0.03; 0.06  1.82 | 0.03  0.03; 0.04  1.63 |
| Number of restorations | 2.02  1.38; 2.75  1.99 | 2.13  1.55; 2.89  1.87 | 0.98  0.76; 1.23  1.63 | 1.07  0.85; 1.33  1.57 | 0.45  0.37; 0.56  1.54 | 0.45  0.40; 0.55  1.37 |

1= multilevel Bayesian regression models were adjusted for age, gender, origin, municipality/region, the highest educational attainment, income percentile (2019), diabetes status, sum of years with 1) dental examination, 2) supragingival care, 3) subgingival care, 4) periodontal surgery, 5) individual preventive service, 6) endodontic treatment, or 6) bitewing radiographs, the sum of 1) restorations and 2) extractions from 2009 to the individual outcome window, and the sum of annual income percentiles between 2009 and 2018.

2= 3^rd^ quartile (Q3) divided by 1^st^ quartile (Q1)


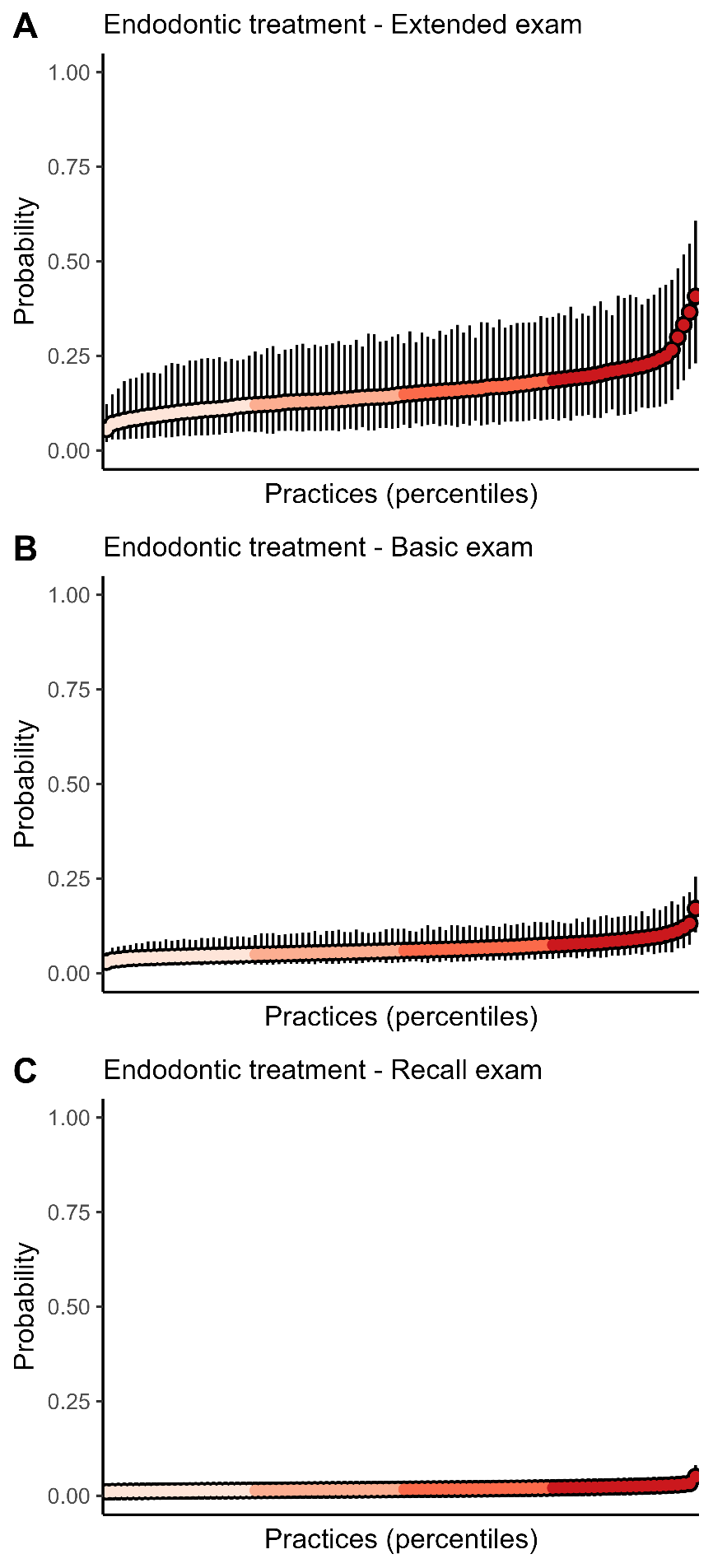


Figure S1. Average predicted probability of endodontic treatment with 95% credibility intervals across practice percentiles for each type of examination (A–C). Predictions were generated using multilevel Bayesian regression models, with individual-level covariates held at their mean or modal values.
